# Improving Physical Health Knowledge of Mental Health Nurses on an Organic Old Age Psychiatry Ward, Woodlands Unit, RDASH, Rotherham site

**DOI:** 10.1192/bjo.2024.299

**Published:** 2024-08-01

**Authors:** Lauren Hartley, Femi Osukoya

**Affiliations:** 1 Rotherham Teaching Hospitals, Rotherham, United Kingdom; 2 Sheffield Teaching Hospitals, Sheffield, United Kingdom

## Abstract

**Aims:**

1. To improve the confidence of the nursing team in ensuring initial assessment and escalation of physical health concerns on an organic old age psychiatry ward, Glade ward, Woodlands unit, RDASH Rotherham.
2. To equip them with the knowledge needed to recognise and promptly escalate concerns about physical health to medics.
3. To foster the relationship between the nursing team and medics to facilitate communication between both teams for the improvement of physical health care of mental health patients.

**Methods:**

Eight weekly teaching sessions were organised and delivered by FY1 and CT1, with each session lasting 10–30 minutes. Short 10-minute teachings followed by questions and answers. Topics were at the request of nursing staff and included physical observations, sepsis, head injury etc.

Attendees included members of the nursing team and allied health professionals (ward managers, mental staff nurses, nursing assistants, student nurses, pharmacy technicians etc.).

Post-Teaching questionnaires filled out after each session rating understanding before and after teaching.

Topics included the commonest physical health conditions on old age mental health wards, including physical observations monitoring and interpretation.

One overall feedback questionnaire was also obtained at the end of all sessions.

**Results:**

Participants emphasised improvement in their level of knowledge and confidence in spotting signs and symptoms as well as derangements in all topics covered.

They reported feeling more included and heard as a member of the team, feeling more confident to escalate abnormal findings to ensure patient reviews. This is evidenced by comments and ratings on feedback forms.

All respondents believed that the teaching sessions should continue as 87.5% felt they were very helpful, while the remaining 12.5% rated it reasonably helpful (4/5).

**Conclusion:**

While the physical health aspect of patients may be easy to overlook or neglect in mental health settings, continuous creation of awareness through interactive teaching sessions can improve staff knowledge and confidence. We need to re-emphasize the importance of a good working relationship between the nursing team and medics to improve the physical health of our patients (while caring for their mental health) and ultimately ensure patient safety at all times.

